# Supplementary material for: Modeling glioblastoma heterogeneity as a dynamic network of cell states
Source: Mol Syst Biol. 2021 Sep 16;17(9):e10105. doi: 10.15252/msb.202010105 (PMC8444284; doi:10.15252/msb.202010105)
Supplement: Supplementary file 5 — Source Data for Figure 3 [file MSB-17-e10105-s001.zip › Figure3A_sourcedata/GSEA_3065/hallmarks_state2.GseaPreranked.1623416269049/HALLMARK_MYOGENESIS.html]

Details for gene set HALLMARK\_MYOGENESIS[GSEA]

|  || Dataset | state2 |
| Phenotype | NoPhenotypeAvailable |
| Upregulated in class | na\_neg |
| GeneSet | HALLMARK\_MYOGENESIS |
| Enrichment Score (ES) | -0.6313162 |
| Normalized Enrichment Score (NES) | -2.4407175 |
| Nominal p-value | 0.0 |
| FDR q-value | 0.0 |
| FWER p-Value | 0.0 |
Table: GSEA Results Summary

  

Fig 1: Enrichment plot: HALLMARK\_MYOGENESIS      
 Profile of the Running ES Score & Positions of GeneSet Members on the Rank Ordered List

  

| PROBE | GENE SYMBOL | GENE\_TITLE | RANK IN GENE LIST | RANK METRIC SCORE | RUNNING ES | CORE ENRICHMENT || 1 | CKB |  |  | 647 | 0.157 | -0.0523 | No |
| 2 | TPM3 |  |  | 694 | 0.151 | -0.0408 | No |
| 3 | FDPS |  |  | 1297 | 0.098 | -0.0947 | No |
| 4 | PKIA |  |  | 1448 | 0.090 | -0.1010 | No |
| 5 | MYL6B |  |  | 1866 | 0.070 | -0.1381 | No |
| 6 | BAG1 |  |  | 2120 | 0.060 | -0.1586 | No |
| 7 | CNN3 |  |  | 2349 | 0.052 | -0.1773 | No |
| 8 | SORBS3 |  |  | 2390 | 0.051 | -0.1761 | No |
| 9 | BIN1 |  |  | 2492 | 0.048 | -0.1817 | No |
| 10 | TEAD4 |  |  | 2546 | 0.046 | -0.1824 | No |
| 11 | SMTN |  |  | 2590 | 0.045 | -0.1821 | No |
| 12 | FHL1 |  |  | 2686 | 0.042 | -0.1877 | No |
| 13 | IFRD1 |  |  | 2840 | 0.038 | -0.2000 | No |
| 14 | CRAT |  |  | 2856 | 0.038 | -0.1975 | No |
| 15 | PDLIM7 |  |  | 3015 | 0.033 | -0.2108 | No |
| 16 | MEF2A |  |  | 3099 | 0.032 | -0.2163 | No |
| 17 | FGF2 |  |  | 3688 | 0.021 | -0.2770 | No |
| 18 | DAPK2 |  |  | 4185 | 0.012 | -0.3287 | No |
| 19 | REEP1 |  |  | 4225 | 0.012 | -0.3316 | No |
| 20 | HDAC5 |  |  | 4227 | 0.011 | -0.3305 | No |
| 21 | SIRT2 |  |  | 4480 | 0.008 | -0.3566 | No |
| 22 | MAPK12 |  |  | 4882 | 0.001 | -0.3994 | No |
| 23 | EFS |  |  | 4894 | 0.001 | -0.4004 | No |
| 24 | FLII |  |  | 5181 | -0.003 | -0.4307 | No |
| 25 | PFKM |  |  | 5382 | -0.006 | -0.4514 | No |
| 26 | MYO1C |  |  | 5468 | -0.008 | -0.4597 | No |
| 27 | ABLIM1 |  |  | 5605 | -0.010 | -0.4731 | No |
| 28 | LARGE1 |  |  | 5611 | -0.010 | -0.4725 | No |
| 29 | KIFC3 |  |  | 5731 | -0.012 | -0.4840 | No |
| 30 | SSPN |  |  | 5862 | -0.014 | -0.4964 | No |
| 31 | GABARAPL2 |  |  | 5891 | -0.015 | -0.4978 | No |
| 32 | ITGB5 |  |  | 6093 | -0.018 | -0.5173 | No |
| 33 | AKT2 |  |  | 6104 | -0.018 | -0.5164 | No |
| 34 | SCHIP1 |  |  | 6274 | -0.021 | -0.5322 | No |
| 35 | NAV2 |  |  | 6389 | -0.023 | -0.5419 | No |
| 36 | MAPRE3 |  |  | 6739 | -0.030 | -0.5760 | No |
| 37 | SH2B1 |  |  | 6773 | -0.031 | -0.5761 | No |
| 38 | FKBP1B |  |  | 6791 | -0.031 | -0.5745 | No |
| 39 | MEF2C |  |  | 6861 | -0.033 | -0.5783 | No |
| 40 | KCNH2 |  |  | 6934 | -0.035 | -0.5823 | No |
| 41 | AEBP1 |  |  | 7162 | -0.041 | -0.6021 | No |
| 42 | SPEG |  |  | 7350 | -0.046 | -0.6171 | No |
| 43 | ACSL1 |  |  | 7456 | -0.049 | -0.6231 | No |
| 44 | EPHB3 |  |  | 7534 | -0.051 | -0.6257 | Yes |
| 45 | PSEN2 |  |  | 7558 | -0.052 | -0.6226 | Yes |
| 46 | DMPK |  |  | 7599 | -0.053 | -0.6211 | Yes |
| 47 | OCEL1 |  |  | 7617 | -0.054 | -0.6171 | Yes |
| 48 | SYNGR2 |  |  | 7633 | -0.054 | -0.6128 | Yes |
| 49 | AGL |  |  | 7637 | -0.054 | -0.6072 | Yes |
| 50 | ADCY9 |  |  | 7646 | -0.055 | -0.6022 | Yes |
| 51 | SOD3 |  |  | 7706 | -0.057 | -0.6024 | Yes |
| 52 | CHRNB1 |  |  | 7708 | -0.057 | -0.5963 | Yes |
| 53 | APOD |  |  | 7757 | -0.058 | -0.5952 | Yes |
| 54 | RIT1 |  |  | 7880 | -0.062 | -0.6015 | Yes |
| 55 | DMD |  |  | 7987 | -0.067 | -0.6056 | Yes |
| 56 | SLC6A8 |  |  | 8016 | -0.068 | -0.6012 | Yes |
| 57 | TPM2 |  |  | 8019 | -0.068 | -0.5941 | Yes |
| 58 | LPIN1 |  |  | 8050 | -0.069 | -0.5898 | Yes |
| 59 | GAA |  |  | 8060 | -0.069 | -0.5832 | Yes |
| 60 | ERBB3 |  |  | 8230 | -0.079 | -0.5928 | Yes |
| 61 | TSC2 |  |  | 8259 | -0.080 | -0.5871 | Yes |
| 62 | ITGB4 |  |  | 8365 | -0.086 | -0.5890 | Yes |
| 63 | TGFB1 |  |  | 8497 | -0.095 | -0.5926 | Yes |
| 64 | CRYAB |  |  | 8519 | -0.098 | -0.5843 | Yes |
| 65 | SORBS1 |  |  | 8562 | -0.101 | -0.5779 | Yes |
| 66 | NCAM1 |  |  | 8660 | -0.108 | -0.5766 | Yes |
| 67 | TAGLN |  |  | 8662 | -0.108 | -0.5650 | Yes |
| 68 | PDE4DIP |  |  | 8665 | -0.108 | -0.5535 | Yes |
| 69 | WWTR1 |  |  | 8752 | -0.117 | -0.5500 | Yes |
| 70 | NOTCH1 |  |  | 8763 | -0.118 | -0.5384 | Yes |
| 71 | MYH9 |  |  | 8778 | -0.119 | -0.5270 | Yes |
| 72 | ADAM12 |  |  | 8799 | -0.122 | -0.5159 | Yes |
| 73 | BHLHE40 |  |  | 8804 | -0.123 | -0.5030 | Yes |
| 74 | PLXNB2 |  |  | 8819 | -0.125 | -0.4909 | Yes |
| 75 | DTNA |  |  | 8828 | -0.126 | -0.4781 | Yes |
| 76 | NQO1 |  |  | 8837 | -0.127 | -0.4652 | Yes |
| 77 | MYLK |  |  | 8862 | -0.131 | -0.4536 | Yes |
| 78 | SPTAN1 |  |  | 8923 | -0.138 | -0.4451 | Yes |
| 79 | SVIL |  |  | 8981 | -0.146 | -0.4354 | Yes |
| 80 | SCD |  |  | 8982 | -0.146 | -0.4195 | Yes |
| 81 | ITGA7 |  |  | 9011 | -0.150 | -0.4063 | Yes |
| 82 | EIF4A2 |  |  | 9067 | -0.160 | -0.3948 | Yes |
| 83 | PRNP |  |  | 9068 | -0.161 | -0.3774 | Yes |
| 84 | COL4A2 |  |  | 9069 | -0.161 | -0.3599 | Yes |
| 85 | COL6A2 |  |  | 9116 | -0.170 | -0.3464 | Yes |
| 86 | ATP6AP1 |  |  | 9160 | -0.179 | -0.3316 | Yes |
| 87 | AGRN |  |  | 9184 | -0.185 | -0.3140 | Yes |
| 88 | GSN |  |  | 9238 | -0.206 | -0.2974 | Yes |
| 89 | IGFBP7 |  |  | 9295 | -0.229 | -0.2786 | Yes |
| 90 | CDKN1A |  |  | 9301 | -0.230 | -0.2542 | Yes |
| 91 | SPARC |  |  | 9384 | -0.332 | -0.2270 | Yes |
| 92 | VIPR1 |  |  | 9385 | -0.332 | -0.1911 | Yes |
| 93 | ITGB1 |  |  | 9388 | -0.339 | -0.1545 | Yes |
| 94 | APP |  |  | 9412 | -0.426 | -0.1109 | Yes |
| 95 | IGFBP3 |  |  | 9424 | -0.502 | -0.0577 | Yes |
| 96 | CLU |  |  | 9430 | -0.547 | 0.0011 | Yes |
Table: GSEA details [plain text format]

  

Fig 2: HALLMARK\_MYOGENESIS: Random ES distribution      
 Gene set null distribution of ES for **HALLMARK\_MYOGENESIS**
